# A pilot study of FDG PET/CT detects a link between brown adipose tissue and breast cancer

**DOI:** 10.1186/1471-2407-14-126

**Published:** 2014-02-25

**Authors:** Qi Cao, Jerome Hersl, Hongloan La, Mark Smith, Jason Jenkins, Olga Goloubeva, Vasken Dilsizian, Katherine Tkaczuk, Wengen Chen, Laundette Jones

**Affiliations:** 1 Departments of Diagnostic Radiology and Nuclear Medicine, University of Maryland School of Medicine, 21201 Baltimore, MD, USA; 2 Department of Pharmacology, University of Maryland School of Medicine, 21201 Baltimore, MD, USA; 3 The University of Maryland Marlene and Stewart Greenebaum Cancer Center, 21201 Baltimore, MD, USA; 4 Department of Epidemiology and Public Health, University of Maryland School of Medicine, 21201 Baltimore, MD, USA

**Keywords:** Breast cancer, Brown adipose tissue, FDG PET/CT

## Abstract

**Background:**

Breast cancer is the second most lethal cancer in women. Understanding biological mechanisms that cause progression of this disease could yield new targets for prevention and treatment. Recent experimental studies suggest that brown adipose tissue (BAT) may play a key role in breast cancer progression. The primary objective for this pilot study was to determine if the prevalence of active BAT in patients with breast cancer is increased compared to cancer patients with other malignancies.

**Methods:**

We retrospectively analyzed data from 96 breast cancer patients who had FDG PET/CT scan for routine staging at the University of Maryland and 96 age- and weight-matched control female patients with other malignancies (predominantly colon cancer) who had undergone FDG PET/CT imaging on the same day. Data on the distribution (bilateral upper neck, supraclavicular and paraspinal regions) and intensity (SUVmax) of active BAT were evaluated by 2 Nuclear Medicine physicians, blinded to the clinical history.

**Results:**

We found sufficient evidence to conclude that based on our sample data the prevalence of active BAT in breast cancer patients’ group is significantly different from that in the control group. The estimated frequency of BAT activity was 3 fold higher in breast cancer patients as compared to controls with other cancers, (16.7% vs. 5.2%, respectively, p = 0.019). When patients were stratified by age in order to determine the possible impact of age related hormonal changes on active BAT among the younger women (≤ 55 years of age), 25.6% breast cancer patients exhibited BAT activity compared to only 2.8% in control women (p = 0.007). In contrast, among the older women (> 55 years of age), the prevalence of active BAT was similar among breast cancer and control women (10.7% vs 6.7%).

**Conclusions:**

In breast cancer patients prevalence of BAT activity on FDGPET/CT is 3-fold greater than in age- and body weight-matched patients with other solid tumor malignancies; this difference is particularly striking among younger women aged < =55. In summary, our retrospective clinical data provide support to pursue prospective clinical and translational studies to further define the role of BAT in breast cancer development and progression.

## Background

Breast cancer is the second most lethal cancer in women [1]. Early stage breast cancers that are confined to the layer of cells in the breast lobule or duct where they originated have a better prognosis and can be cured [1]. However, the prognosis for advanced stage cancers that have invaded locally and spread to other parts of the body is poor, with median survival averaging 18–24 months [1]. Understanding biological mechanisms that could affect the possible progression of breast cancer from the high risk lesions such as atypical ductal or lobular hyperplasia to noninvasive cancers, ductal and lobular carcinoma in situ to invasive cancers could yield new targets for prevention of breast cancer and treatment of more advanced stages and ultimately improve patient survival.

Once considered inert, adipose tissue has emerged as a key player in cancer development and progression [2]. Adipocyte-derived factors have been shown to both stimulate or inhibit cell growth and cause systemic inflammation [2]. This knowledge has sparked great interest in cancer researchers worldwide to focus on the link between obesity or dysfunctional white adipose tissue (WAT) and cancer [2-7]. Yet another type of fat, brown adipose tissue (BAT), has recently emerged as an interest in context of cancer and tumor development as well [4,5]. BAT is characterized by its multilocular cells, numerous mitochondria, and high vascularity, functions to dissipate energy as heat in response to cold temperatures [8]. Studies in mice have shown that the activation of BAT is associated with the synthesis and secretion of angiogenic and growth factors, resulting in markedly increased vascular density due to activation of angiogenesis [9,10]. Therefore, research investigating the potential role of active BAT in adults should also be accounted for in the context of cancer and tumor development.

Data on connections of BAT with neoplasms of nonadipocytic origin is limited. In humans, BAT was considered only to be of significance in infants as a source of easily accessible energy that would regress and gradually be replaced by WAT with age [8]. Yet, emerging studies point to a potential association of specific BAT features to certain mutated tumor suppressor genes [4,11]. In particular, we observed a significantly increased deposition of BAT in the adult mammary fat pad of a mouse model of *Breast Cancer gene 1 (BRCA1)* breast cancer compared to age-matched mammary glands from wildtype mice [11]. This was quite abnormal considering that high amounts BAT are usually only in the mammary gland from birth until about 8–10 weeks of age (near the completion of puberty) [12,13]. Considering that high amounts of BAT are exclusively detected in the mouse early in mammary gland development, particularly during stages of ductal growth and increased estrogen signaling, one can speculate that BAT may play a role in mammary ductal growth. Moreover, the high level of BAT in the mammary gland of these mice was associated with increased angiogenesis [11]. Indeed it is known that angiogenesis is an essential step for breast cancer progression and dissemination [14]. Therefore, the unexpected finding of the sustained BAT phenotype in the mammary gland of adult mice predisposed to breast cancer raises an important issue of whether there is a relationship between high levels of BAT and breast cancer. Although such a possibility has not been explored clinically, this is an important next step to determine the value and relevance of our findings for human breast cancer.

18 F-fluorodeoxyglucose (FDG) Positron Emission Tomography/Computerized Tomography (PET/CT) is a widely used modality of imaging glucose metabolism in cancer cells for staging of primary cancer and detection of distant metastasis in patients [15,16]. Due to its similar biological properties of hypermetabolism to cancer cells, BAT may also have intense FDG uptake and can be as a “false-positive” for cancer evaluation if detected on PET/CT [15-17]. In the current, study we used FDG PET/CT as a non-invasive approach to determine whether there is a significant difference between FDG uptake in BAT between patients with breast cancer and those scanned for other malignancies. The primary objective for this retrospective pilot study was to explore the patterns of BAT activity with FDG PET/CT in patients with breast cancer with a broader aim to form a basis for further studies that will help to determine whether BAT may be an important determinant for breast cancer risk and progression. We also provide an appreciation of the site-based distribution of BAT observed in the images and the clinical and pathological characteristics of the groups of patients in this study.

## Methods

### Patients

This retrospective image and chart/medical record review study was approved by the University of Maryland Baltimore Institutional Review Board (IRB). The Institutional review board waived the need to obtain consent from patients. Patients were undergoing their FDG PET/CT scans as part of routine standard of care and no changes to standard of care were made. We reviewed the distribution and intensity (maximum standard uptake value, SUVmax) of BAT on FDG PET/CT scans in a total of 96 breast cancer patients who had PET/CT scans from October 2010 to September 2012 in our institution. If a patient had multiple PET/CT scans during this interval, only the first PET/CT scan was analyzed to avoid over counting BAT rate in the same patient. For comparison, each breast cancer patient was assigned a paired-control of a non breast cancer patient (mainly colon cancer) who had a PET/CT scan on the same day. Both groups were carefully matched in reference to sex (all female), age (± 5 years), and body weight (± 5 kg) to control for some of the known factors that could potentially affect FDG uptake in BAT.

### FDG PET/CT imaging

Patients fasted for at least 4 h before PET/CT imaging and had a measured finger stick glucose level less than 220 mg/dl before the administration of FDG. As routine practice, patients with fasting blood glucose level greater than 220 mg/dl were excluded from study as high glucose may interfere with 18 F-FDG tracer uptake based on the imaging guidelines. Scans were acquired approximately 60 min after the injection of about 555 MBq (15 mCi) FDG with the Gemini PET/CT (Philips Medical Systems, Cleveland, Ohio, USA) scanner with a 16-slice Brilliance CT.

### Data collection

The distribution of the BAT was analyzed in the breast cancer patients (n = 96) and the non-breast cancer patients (n = 96) with one scan per each patient by 2 nuclear medicine physicians blinded to the clinical history. Training and expertise of the two nuclear medicine physicians reading FDG PET/CT was 7 years and 3 years, respectively, with an interpersonal variation of 0. The interpretation of a positive active BAT site on PET/CT was based on the imaging findings of focal FDG uptake in adipose tissue that is visually more intense than the surrounding muscle activity, which is simple “yes” or “no” with no case showing equivocal findings. No SUVmax threshold value was set to define a positive BAT. The location of the BAT was recorded in the bilateral neck, supraclavicular and paraspinal regions. The SUVmax of brown fat was measured, a positive BAT case was defined as at least one positive BAT site in any of the locations in the neck, supraclavicular or paraspinal regions.

### Statistical analysis

The prevalence of positive BAT cases on FDG PET/CT in the breast cancer patients was estimated and compared to the control patients using the Fisher’s exact test for 2×2 tables. The Fisher-Freeman-Halton test with Monte-Carlo simulations was applied for rxc contingency tables. Plausible risk factors were also estimated using the logistic regression approach. The distribution of BAT was compared using the non-parametric approach, the Wilcoxon test. The ANOVA method was applied to assess the differences of SUVmax of BAT in different locations between the 2 study groups. All statistical tests were two-sided and done at the 0.05 level of significance. Analyses were conducted in SAS (SAS Institute Inc., Cary, NC, v.9.3).

## Results and discussion

### Study population

Generally BAT occurrence on FDG PET/CT is thought to be related to several factors, inclucing sex, age and temperature [8,9]. Notably, BAT is inversely correlated with obesity and body mass index (BMI) [8,9]. To control these factors, in the current study, we assigned each breast cancer patient a paired control with an FDG PET/CT scan done on the same day for other cancers (mainly colon cancer), matched with sex, age and body weight. The BAT prevalence rate is 5.2% in the control group in the current study, similar to the reported 2.3-10% in the general population [8,9]. The non breast cancer patients (n = 96) consisted of colorectal cancer (n = 54), lung cancer (n = 13), lymphoma (n = 12), head neck cancer (n = 7), pancreatic cancer (n = 6) and others (n = 4, GIST 2, leiomyosarcoma 1, and carcinoid 1). Mean age was 58.6 ± 11.4 in the breast cancer and 58.2 ± 12.3 in the controls, and mean body weight (kg) was 66.4 ± 9.6 in the breast cancer and 70.0 ± 10.8 in the controls. There were no significant differences between the mean ages in the subgroups: [age ≤ 55 yrs: Breast cancer 46.3 +/− 6.7 vs. Non breast cancer 47.9 ± 6.5, p = 0.288] and [age > 55 yrs: Breast cancer 65.9 ± 8.3 vs. Non breast cancer 66.1 ± 7.4, p = 0.878].

### Relationship between levels of BAT and breast cancer

**Figure 1:**
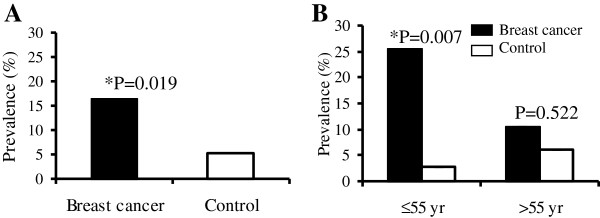
**Prevalence of BAT case in the breast cancer and control patients. (A)** Expression of BAT was compared in the 96 breast cancer patient and 96 paired control patients with other cancers, using exact Fisher’s test. A positive case is defined as at least 1 site of BAT in any location of the neck, supraclavicular and paraspinal region. **(B)** Stratification of the brown fat prevalence by age of ≤ 55 and > 55 years old to roughly divide the patients into pre- and post-menopausal groups.

In adults, BAT is thought to normally remain inactive and thus does not appear on FDG PET/CT unless stimulated, such as exposure to cold temperature (15–17). In the current clinical study, we demonstrate that the prevalence of metabolically active BAT as seen on FDG PET/CT is higher in breast cancer patients than their paired controls with other malignancies. Among the breast cancer group, there was 16/96 (16.7%) BAT positive cases compared to only 5/96 (5.2%) positive cases among the non breast cancer control group. The prevalence of BAT in the breast cancer group was about 3-fold higher than that in the non breast cancer control group (p = 0.019) (Figure 1A). To find out if possible changes in age related sex hormone levels may have an effect on BAT, we stratified the data by age (≤ 55 and > 55 ) to approximately divide the subjects into pre and post-menopausal groups. We found that the difference in BAT prevalence associated with ≤ 55 years old patients was greater in the breast cancer group (10/39 = 25.6%) than in the non breast cancer control (1/36 = 2.8%) (p = 0.007). In contrast, among those who were > 55 years old, the difference in BAT prevalence in the breast cancer group (6/57 = 10.5%) and the non breast cancer control group (4/60 = 6.7%) (p = 0.522) was no longer observed (Figure 1B). Although we are not able to obtain each patient’s history of menopausal status given the retrospective nature of our study, the findings of age related increase in BAT uptake suggest that sex hormone levels may play a role in the development of brown fat and breast cancer.

There are two other studies that have indicated high levels of 18 F-FDG uptake in breast cancer populations. In one small retrospective study, Rousseau et al. used PET/CT to evaluate early response to neoadjuvant chemotherapy for breast cancer in 33 patients with breast cancer [18]. They reported that 35% of PET/CT images (12 out of 33) from breast cancer patients were positive for active metabolism in BAT [18]. Most recently in a larger retrospective study, Cronin *et al*., studied the prevalence of BAT in patients with different types of cancer and found that the prevalence of active BAT was highest in patients with breast cancer and sarcomas [19]*.* To the best of our knowledge, this is the first that directly compared the prevalence of active BAT in breast cancer patients with their paired age- and weigh-matched controls. We acknowledge that without immunohistochemical confirmation, FDG PET/CT may markedly underestimate true prevalence of BAT. Thus, it is unknown whether FDG PET underestimates true prevalence of BAT or not in normal or cancer patients. Even so, this “underestimation” applies to both the cancer and control groups.

Mechanisms for a potential association between active BAT and breast cancer are not entirely clear and currently under investigation. We suggest two alternate hypotheses. First, BAT could participate as an active participant in the progression of breast cancer. Given its high vascularity and ability to secrete bioactive molecule [9,10]. BAT could potentially cause nearby pre-cancerous epithelial cells to proliferate more rapidly, accelerating the progression of breast cancer. Alternatively, BAT could participate as a passive participant in the progression of breast cancer. Considering cancer cells are also known to secrete bioactive molecules to support its own progression [20], activation of BAT could be secondary to the breast cancer.

### Site-based distribution of BAT and clinical and pathological characteristics

The site distribution of BAT found in the common distinct anatomical locations among the patient groups are reported in Table 1. There was no difference in the distribution of BAT in the 6 locations of the left and right neck, left and right supraclavicular, and paraspinal regions between the breast cancer and the non breast cancer control groups (Table 1). In addition, no difference of BAT FDG uptake (mean of SUVmax) was found in each of the locations or in any of the sites between the 2 groups. Previously, we had shown that there were significant differences in the deposition of BAT in the adult mammary fat pad of a mouse model of breast cancer compared to age-matched mammary glands from wildtype mice [12].

**Table 1:** Site-based distribution of brown fat seen on FDG PET/CT in the breast cancer and controls

	**Neck**		**Supraclavicular region**		**Paraspinal region**		**Total**
	**Left**	**Right**	**Left**	**Right**	**Left**	**Right**	
Breast cancer	4	4	10	9	12	8	47
Control	3	2	3	3	2	1	14
Total	7	6	13	12	14	9	61

In contrast to rodents, there is only limited understanding of the distribution of BAT within the human breast. Multilocular adipocytes resembling brown adipocytes have been detected in postmortem human infant breasts. [21,22]. Albeit rare, the observation of brown fat tumors (hibernomas) in the breast have been described [23-27]. We also acknowledge that activation of BAT tissue within the breasts in female patients undergoing FDG PET/CT is typically not seen. This could be due to the heterogeneity of cell types within the breast compared to the homogenous cell types in BAT. Interestingly, studies have shown that exposure of humans and rodents to cold activates thermogenic activity in brown adipose tissue (BAT) [10,28]. Biopsies from mice also show that this BAT activation causes an obvious transition from subcutaneous white adipose tissue (WAT) into brown-like adipose tissue (BRITE or BEIGE). If this phenomenon also occurs in humans, than the breast cancer patients positive for active BAT in common regions are more likely to show brown-like adipose tissue within their subcutaneous breast adipose tissue biopsies. However, in the current study, we did not explore this hypothesis. Therefore, it would be most ideal to examine breast tissue slides from patient biopsies by H&E or for immunohistochemical markers for BAT to determine if there are cells that resemble the morphology and molecular characteristics of brown adipocytes, respectively.

Finally, we assessed whether there was a relationship between BAT and disease stage, histological type and tumor stage in breast cancer patients. The available clinical and pathologic data are reported in Table 2. Although significant differences in the clinical and tumor pathological characteristics (stage, histologic grade, estrogen receptor (ER), progesterone receptor (PR), *BRCA1* status) could not be determined in this retrospective study, we do see higher prevalence of Human Epidermal Growth Factor Receptor 2 (HER2) overexpression in BAT positive breast cancer cases. Notably, in the breast cancer group, the BAT positive patients tend to have a 2-fold higher HER2 positive rate (6/16 = 37.5%) than the BAT negative patients (12/96 = 15.0%) (P = 0.06), but this did not reach statistical significance.

**Table 2:** Clinical and pathological characteristics in breast cancer patients with and without brown fat

	**Brown fat +**	**Brown fat +**
Race		
White	7 (43.8)	38 (47.5)
Black	6 (37.5)	39 (48.8)
Other	3 (18.7)	3 ( 3.7)
Tumor size (cm)	3.26 ± 1.85	3.38 ± 1.74
Tumor histology (%)		
Ductal	14 (87.5)	59 (73.8)
Adeno	0 (0.0)	1 ( 1.3)
Lobular	0 (0.0)	9 (11.3)
Unknown	2 (12.5)	11 (13.7)
Nodal metastasis		
Positive	8 (50.0)	32 (40.0)
Negative	3 (18.8)	17 (21.3)
Unknown	5 (31.2)	36 (45.0)
Distant metastasis		
Positive	8 (50.0)	44 (55.0)
Negative	8 (50.0)	36 (45.0)
Stage		
I	0	4 ( 5.0)
II	3 (18.8)	15 (18.8)
III	3 (18.8)	9 (11.3)
IV	10 (52.5)	45 (56.3)
Unknown	0	7 ( 8.7)
ER		
Positive	10 (62.5)	42 (52.5)
Negative	4 (25.0)	27 (33.8)
Unknown	2 (12.5)	11 (13.8)
PR		
Positive	7 (43.8)	36 (45.0)
Negative	6 (37.5)	33 (41.3)
Unknown	3 (18.7)	11 (13.7)
HER2		
Positive	6 (37.5)	12 (15.0)
Negative	7 (43.8)	54 (67.5)
Unknown	3 (18.7)	14 (17.5)
Grade		
I	0	4 ( 5.0)
II	5 (31.3)	15 (18.8)
III	7 (43.8)	28 (35.0)
Unknown	4 (25.0)	33 (41.2)

Multiple factors contributing to FDG uptake in BAT have been described in the literature [16,17,20]. While care was taken to control for some of the known factors that could potentially affect FDG uptake in BAT including matching study participants for age, sex, weight, and same day of scan between groups, due to the retrospective nature of this study, complete clinical data were not available for each patient. Therefore, we acknowledge several limitations to this study including the unavailability of the actual menopause status, and the timing of prior medication and treatment history of every patient. Additionally, considering the majority of the control patients had colorectal cancer, it is important to note that obesity is a well known risk factor for colorectal cancer [29]. Lower prevalence of BAT tissue in patients with colon cancer may be related to obesity because subjects with BAT tend to be those who are leaner [29]. Thus, while both breast cancer and control group were matched in reference to body weight, it does not measure obesity making it a possible confounding variable in this study. We recognize that in our study, the uptake tissue was not biopsied to validate the presence of BAT. Therefore, FDG PET/CT could potentially underestimate or overestimate the true prevalence of BAT. Nevertheless, the correlation of uptake in hypermetabolic BAT is well-supported and a recognized feature of FDG PET. Further, we anticipate that any “underestimation or overestimation” would apply to both the cancer and control groups [15-19]. In the future, it will be worthwhile to conduct a prospective study in larger patient population. This would afford the opportunity to collect information and samples from all subjects thereby potentially eliminating some of the confounding variables in the evaluation of the results.

## Conclusions

We have conducted a retrospective investigation using non-invasive FDG PET/CT imaging to explore the relationship between levels of BAT presence and breast cancer in adult women. Early indications show that there is an increased prevalence of metabolically active BAT seen on FDG PET/CT in breast cancer patients compared to their pair matched control patients with other cancers. This finding is most prevalent in younger premenopausal patients, indicating a possible role of sex hormones. These clinical data provide further support to our experimental studies that BAT is associated with breast cancer, however further studies are required to clarify a potential mechanism. We believe that our investigations to determine whether BAT plays an active or passive role in breast cancer progression will provide future insight on whether breast cancer patients who are positive for FDG uptake in BAT are likely to have more or less aggressive tumors than those patients that do not.

## Abbreviations

ANOVA: Analysis of variance; BMI: Body mass index; BRCA1: Breast cancer gene-1; BAT: Brown adipose tissue; CI: Confidence interval; ER: Estrogen receptor; FDG: Fluorodeoxyglucose; HER2: Human epidermal growth factor receptor 2; PET/CT: Positron emission tomography/computerized tomography; PR: Progesterone receptor; SUVmax: Maximum standard uptake value.

## Competing interests

The authors declare that they have no competing interests.

## Authors’ contributions

QC participated in the acquisition of data for the PET/CT scans, and in the interpretation of the data. JH and HL participated in the acquisition of patient information, analysis and interpretation of the data. MS and JJ participated in the acquisition and imaging process of the PET/CT scans. KT and WC participated in the design of the study, provided guidance throughout the study process, participated in the analysis and interpretation of the data and helped draft the manuscript. OG participated in the design of the study and performed the statistical analysis. VD supervised all aspects of the PET/CT data acquisition and data analysis and contributed to the manuscript preparation. LJ conceived of the study, and participated in its design and interpretation of data, the coordination of the Nuclear Medicine, Oncology and Pharmacology Departments and contributed to the manuscript preparation. All authors were involved in revising the manuscript, and have read and approved the final manuscript.

## Pre-publication history

The pre-publication history for this paper can be accessed here:

http://www.biomedcentral.com/1471-2407/14/126/prepub
